# HIF-1α promoted vasculogenic mimicry formation in hepatocellular carcinoma through LOXL2 up-regulation in hypoxic tumor microenvironment

**DOI:** 10.1186/s13046-017-0533-1

**Published:** 2017-04-27

**Authors:** Meili Wang, Xiulan Zhao, Dongwang Zhu, Tieju Liu, Xiaohui Liang, Fang Liu, Yanhui Zhang, Xueyi Dong, Baocun Sun

**Affiliations:** 1 Department of Pathology, Tianjin Medical University, Tianjin, 300070 China; 2 Department of Surgery, Stomatological Hospital of Tianjin Medical University, Tianjin, 300070 China; 3 Department of Pathology, General Hospital of Tianjin Medical University, Tianjin, 300052 China; 4 Department of Pathology, Cancer Hospital of Tianjin Medical University, Tianjin, 300060 China

**Keywords:** Hypoxic tumor microenvironment, HIF-1α, LOXL2, Vasculogenic mimicry, EMT, Tumor progression, Hepatocellular carcinoma

## Abstract

**Background:**

The incidence and mortality rates of hepatocellular carcinoma (HCC) have steadily increased in recent years. A hypoxic microenvironment is one of the most important characteristics of solid tumors which has been shown to promote tumor metastasis, epithelial-mesenchymal transition and angiogenesis. Epithelial-mesenchymal transition and vasculogenic mimicry have been regarded as crucial contributing factors to cancer progression. HIF-1α functions as a master transcriptional regulator in the adaptive response to hypoxia. Lysyl oxidases like 2 (LOXL2) is a member of the lysyl oxidase family, which main function is to catalyze the covalent cross-linkages of collagen and elastin in the extracellular matrix. Recent work has demonstrated that HIF-1α promotes the expression of LOXL2, which is believed to amplify tumor aggressiveness. LOXL2 has shown to promote metastasis and is correlated with poor prognosis in hepatocellular carcinoma. The purpose of our study is to explore the role of HIF-1α in progression and metastasis of hepatocellular carcinoma by promoting the expression of LOXL2 as well as the potential regulatory mechanism.

**Methods:**

HIF-1α, LOXL2 expression and CD31/periodic acid-Schiff double staining in HCC patient samples were examined by immunohistochemical staining. shRNA plasmids against HIF-1α was used to determine whether LOXL2 been increased by HIF-1α. We monitored a series of rescue assays to demonstrate our hypothesis that LOXL2 is required and sufficient for HIF-1α induced EMT and VM formation, which mediates cellular transformation and takes effect in cellular invasion. Then we performed GeneChip® Human Transcriptome Array (HTA) 2.0 in HepG2 cells, HepG2 cells overexpressed LOXL2 and HepG2 cells treated with CoCl_2_.

**Results:**

In clinical HCC tissues, it confirmed a positive relationship between HIF-1α and LOXL2 protein. Importantly, HIF-1α and LOXL2 high expression and the presence of vasculogenic mimicry were correlated to poor prognosis. HIF-1α was found to induce EMT, HCC cell migration, invasion and VM formation by regulating LOXL2. The results of microarray assays were analyzed.

**Conclusion:**

HIF-1α plays an important role in the development of HCC by promoting HCC metastasis, EMT and VM through up-regulating LOXL2. This study highlights the potential therapeutic value of targeting LOXL2 for suppression of HCC metastasis and progression.

**Electronic supplementary material:**

The online version of this article (doi:10.1186/s13046-017-0533-1) contains supplementary material, which is available to authorized users.

## Background

Hepatocellular carcinoma (HCC) is one of the most prevalent primary liver malignancies, as both the incidence and mortality rates of HCC have steadily increased in recent years [1]. Although promising treatment strategies have been reported, the dismal outcome and poor median survival remain unchanged. The tumor microenvironment, which provides a supportive framework for the cancer cells, comprises numerous cell types, including cancer-associated fibroblasts, vascular components such as endothelial cells and pericytes, and immune cells such as tumor-associated macrophages, as well as ECM components [2]. Within the tumor microenvironment, hypoxia is the most common phenomenon because of the vast energy and oxygen consumption [3]. Hypoxia contributes to the progression of various cancers by activating adaptive transcriptional programs that promote cell survival, motility, and angiogenesis [4]. The hypoxic microenvironment of tumors affects the metabolism, angiogenesis, and survival of cells orchestrated by hypoxia-inducible factor-1α (HIF-1α) activity [5].

HIF-1α is a crucial transcription factor that contributes to the tumor EMT, which is characterized by the loss of cell adhesion, repression of E-cadherin expression, acquisition of the mesenchymal marker vimentin, and increased cell motility and invasiveness [6]. Our previous study demonstrated that EMT is critical for vasculogenic mimicry (VM), an abnormal blood supply pattern. EMT progression and VM may explain the elevated risk of metastasis, tumor recurrence, and shorter survival period in patients with VM-positive HCC [7].

LOXL2 is a member of the lysyl oxidase (LOX) family, which comprises five members: the prototypical LOX and its four related members LOXL1-4. LOXL2 is a secreted copper-dependent amine oxidase, and its main role is to catalyze the covalent cross-linkages of collagen and elastin in the extracellular matrix (ECM). This occurs through the oxidative deamination of peptidyl lysine residues on components of the ECM [8, 9]. Additionally, several studies have shown that LOXL2 down-regulates E-cadherin expression and promotes the epithelial-mesenchymal transition (EMT) [10–12]. In addition, previous studies have reported that overexpression of LOXL2 promotes invasion and metastasis, thus resulting in its value as a marker of poor prognosis in several tumors [13–16]. However, the role of LOXL2 expression in cancer cells (referred as ‘cancer cell-derived LOLX2’ in this report) in hypoxic tumor microenvironment remains incompletely understood, especially regarding molecular mechanisms.

In our present study, we demonstrate that LOXL2 is a direct target of HIF-1 and that induction of LOXL2 is necessary and sufficient to repress E-cadherin under hypoxic conditions. HIF-1α plays an important role in the development of HCC by promoting HCC metastasis, EMT and VM through up-regulating LOXL2. Futhermore, this study revealed the targets genes of LOXL2 in HCC cells and provide molecular basis of cancer cell-derived LOXL2 functioning with the hypoxia-mimetic agent CoCl_2._

## Methods

### Collection of patient samples

A total of 201 primary tumor specimens were obtained from the Tumor Tissue Bank of the Tianjin Cancer Hospital (Tianjin, China). The HCC specimens were collected from patients who underwent hepatectomy between 2001 and 2009. A diagnosis of HCC in these samples was verified by pathologists. Detailed pathological and clinical data were collected for all the samples, including the Edmondson tumor grade, metastasis and survival duration. The use of these tissue samples was approved by the Institutional Research Committee.

### Immunohistochemistry of tumor samples

Immunohistochemical staining was done to validate the expression of HIF-1α and LOXL2 in HCC tumor tissues. The tissue sections were deparaffinized, hydrated and rehydrated based on standard protocols. Antigen retrieval was performed, and non-specific binding sites were blocked. The sections were then incubated with rabbit monoclonal anti-HIF-1α (ZA-0562, USA) and rabbit polyclonal anti-LOXL2 (1:800, Cat. #GTX105085; GeneTex, California, USA) primary antibodies overnight at 4 °C, and the secondary antibody was incubated with the samples for 30 min at 37 °C. The color was developed using a 3,3’-diaminobenzidine chromogen (DAB) solution.

### CD31/PAS double staining

Immunohistochemical staining with CD31 was performed on the sections as described above prior to PAS staining. Then, the slides were treated with periodic acid solution for 10 min and rinsed with distilled water for 5 min. In a dark chamber, the slides were submerged in Schiff solution for 15 min at 37 °C. After washing the slides under running water for 20 min, all of the sections were counterstained with hematoxylin, dehydrated, and mounted.

### Immunohistochemical scoring

The protein expression levels were quantified according the intensity and percentage of positive tumor cells. At least 10 randomly selected microscope fields per slide were counted with approximately 100 tumor cells per field. The extent of positivity (“extent of distribution” of positive cells) was graded on the following scale: 0 for <10% positive cells, 1 for <25% positive cells, 2 for <50% positive cells, and 3 for more than 50% positive cells. The intensity of the staining was scored on a scale of 0–3 as follows: 0, no appreciable staining in the tumor cells; 1, barely detectable staining in the cytoplasm and/or nucleus compared to the stromal elements; 2, readily visible brown staining; and 3, dark brown staining in tumor cells obscuring the cytoplasm and/or nucleus. The product (staining index) of intensity and percentage scores were utilized to determine the result. For statistical analysis, a total score <4 was defined as negative/low expression, while scores ≥4 were defined as positive/high expression.

### Cell culture and CoCl_2_ treatment

The Bel7402 and HepG2 cell lines were obtained from the American Type Culture Collection. Both these cell lines were cultured in RPMI 1640 and MEM supplemented with 10% fetal bovine serum (FBS; Invitrogen). We used cobalt chloride (CoCl_2_) to mimic hypoxic conditions. Cells were seeded in dishes or plates and grown for 24 h in complete medium. The medium was removed, and cells were washed with PBS. Afterwards, the cells were treated with 150 μM CoCl_2_ and incubated for 48 h.

### Plasmids

HIF-1α and LOXL2 suppression was mediated by lentiviral infection using OmicsLink short hairpin RNA (shRNA) Expression Clones (Catalog no. HSH008831-LVRH1MH and HSH010830-LVRU6GP, GeneCopoeia; indicated in the figures as shHIF-1α and shLOXL2, respectively). CSHCTR001-LVRU6MH and CSHCTR001-LVRU6GP encoding non-specific shRNA were also used as negative controls.

LOXL2 overexpression was achieved by cloning the ORF into a lentiviral vector that induced elevated expression of the LOXL2 gene (Catalog no. EX-Y2020-LV201; indicated in the figures as LOXL2). EX-NEG-Lv201 was used as a negative control. Cells were transduced using a Lenti-Pac HIV Expression Packaging Kit (Catalog no. HPK-LvTR-40) according to the manufacturer’s protocol. Puromycin was used as the selection marker to obtain a stable cell line.

### RNA extraction and quantitative real-time polymerase reaction (qRT-PCR)

Total RNA was extracted using TRIzol reagent (Tiangen Biotech, Beijing, China) according to the manufacturer’s instructions; cDNA was prepared using the PrimeScript™ RT reagent Kit With gDNA Eraser (TaKaRa). Quantitative PCR (qPCR) was performed using the 7500/7500 Fast Real-Time PCR System (Applied Biosystems) with Tli RNaseH Plus (TaKaRa, RR820A). The following primers were used for qRT-PCR. HIF-1α, forward, 5’-GTCGGACAGCCTCACCAAACAGAGC-3’; reverse, 5’-GTTAACTTGATCCAAAGCTCTGAG-3’; LOXL2,forward,5’-CATCTGGATGTACAACTGCCACATA-3’;reverse,5’-AGCCCGCTGAAGTGCTCAA-3’; CDH1,forward, 5’-GAGTGCCAACTGGACCATTCAGT-3’;reverse, 5’-AGTCACCCACCTCTAAGGCCATC-3’; CDH5,forward, 5’-AGCCAGCCCAGCCCTCAC-3’; reverse: 5’CCTGTCAGCCGACCGTCTTTG-3’. Vimentin,forward,5’-TGACATTGAGATTGCCACCTACA-3’;reverse,5’- TCAACCGTCTTATACAGAAGTGTCC-3’. Glyceraldehyde3-phosphate dehydrogenase (GAPDH) was used as an endogenous control (forward primer, 5’-CCTGGCCAAGGTCATCCATGAC-3’; reverse primer, 5’-TGTCATACCAGGA- AATGAGCTTG-3’), and relative fold changes were calculated using the 2^-∆∆Ct^ method.

### Western blot analysis

Whole cell lysates were separated by sodium dodecyl sulfate-polyacrylamide gel electrophoresis and transferred onto polyvinylidene difluoride membranes. Blots were blocked and incubated with antibodies targeting LOXL2 (1:1000, Cat. #GTX105085; GeneTex, California, USA), HIF-1α (1:200, Cat. #Ab1; Abcam, Cambridge, UK), E-cadherin (1:200, Cat. #ZS-7870; Zhongshan Chemical Co, Beijing, China), vimentin (1:1000, Cat. #ab92547; Abcam, Cambridge, UK) and VE-cadherin (1:500, Cat. #ab33168; Abcam, Cambridge, UK). The membranes were then incubated with a secondary antibody (1:2000, Cat. #sc-2055, Cat. #sc-2004, Santa Cruz, CA, USA). The blots were developed using an enhanced chemiluminescence detection kit (Amersham Pharmacia Biotech, Piscataway, NJ, USA). To analyze protein loading, a monoclonal β-actin antibody (1:2000, Cat. #sc-47778; Santa Cruz, CA, USA) was used.

### Immunofluorescence

Cells were plated onto coverslips and fixed in ice cold methanol for 10 min. The cells were blocked with 5% FBS and incubated with the primary antibodies and the FITC-conjugated secondary antibodies. The sections were counterstained with 4,6diamino-2-phenylindole and observed under a fluorescence microscope (80i, Nikon) at × 200 magnification.

### Transwell assay

HepG2 and Bel7402 HCC cells (1x10^5^ cells) in 100 μl of MEM and RPMI 1640 without FBS were seeded into Matrigel matrix-coated upper 24 wells (1 mg/mL; BD Biosciences) containing polyethylene terephthalate filters with 8 μm porosity (Invitrogen). The lower chamber was filled with 10% FBS-containing medium. The cells were incubated for 48 h, and non- invading cells were removed from the upper surface of the membrane. The cells that invaded the Matrigel matrix and adhered to the bottom surface of the membrane were fixed with methanol and stained with 0.5% crystal violet. The number of invading cells was counted using an inverted light microscope (100× magnification) (Nikon). Each experiment was performed in triplicate.

### Wound healing assay

For wound-healing assays, HepG2 and Bel7402 HCC cells were plated in 12-well culture plates. When the cells formed a monolayer, a straight scratch was made in the center of each well using a micropipette tip, and the cells were washed with PBS and incubated in serum-free medium. Cell motility was assessed by measuring the movement of the cells into the scratch in each well. We opened these pictures through drawing software and used scaleplate to measure the length of the wound. The migration rate (MR) was monitored after 24, 48 and 72 h. The following formula was used to calculate MR at different time points: MR = (d − d′)/d, where d is the length of the wound at time 0, and d′ is the length at other different time points. Each experiment was performed in triplicate.

### Three-dimensional culture

In vitro VM vitro was evaluated using a 3D culture system. To create the 3D culture, Matrigel (BD, USA) was thawed at 4°Cand added to each well of a 96-well plate (30 μl/well). The plates were placed on ice and then moved into a 37 °C incubator containing 5% CO_2_ for 12 h to solidify the Matrigel. Tumor cells in complete medium were then seeded onto the gel and incubated at 37 °C for 24 h. The formation of capillary-like structures was observed under phase contrast microscopy (100× magnification). Each experiment was performed in triplicate.

### Human genome-wide expression profiling and bioinformatic analyses

Total RNA from three groups of HCC cells was extracted using TRIzol reagent (Tiangen Biotech, Beijing, China). Briefly, the extracted RNA was labeled and hybridized on an Affymetrix Gene Expression Microarray (design ID: OE2015Q1548; Agilent Technologies, Santa Clara, CA, USA) by Oebiotech Co. (Shanghai, China). Statistical analyses and data normalization were performed using GeneSpring (version 13.1) software (Agilent Technologies). Differentially expressed genes were then identified by observing fold changes as well as by calculating the *P*-values using t-tests. The thresholds set for up-regulated and down-regulated genes were a fold change ≥2.0 and a *P*-value <0.05.

### Statistical analysis

Analysis was performed using SPSS 21.0. The pathological and clinical characteristics of the two groups in hepatocellular carcinoma cases were assessed by the χ^2^test. Mean values were assessed using a two-tailed Student’s t test for paired data. Survival curves were estimated using the Kaplan-Meier method and compared by a log-rank test. Statistical significance was defined as *p* < 0.05.

## Results

### Correlation of HIF-1α and LOXL2 expression as well as VM with the clinicopathological parameters and prognosis of patients with hepatocellular carcinoma

**Fig. 1:**
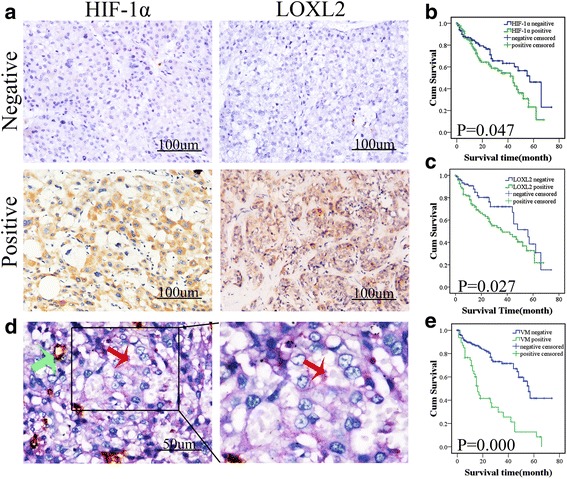
Expression of HIF-1α and LOXL2 correlates with vasculogenic mimicry (VM) and poor prognosis in HCC samples. **a** Hepatocellular carcinoma specimens were analyzed by immunohistochemistry. The first panel for negative expression and the second panel for positive expression of HIF-1α and LOXL2 (×200,bars 100um). **b**, **c** Overall survival of patients with HIF-1α-positive and HIF-1α-negative samples, LOXL2-positive and LOXL2-negative samples. Kaplan–Meier analysis showed that the patients with HIF-1α-positive and LOXL2-positive samples displayed poorer prognosis. **d** CD31/PAS double staining displayed VM channels in hepatocellular carcinoma specimens. The channels (red arrowhead) lined with tumor cells contained red blood cells and were CD31-negative and PAS-positive. The EDVs were CD31-positive (green arrowhead) (×400, bars 50um). **e** Prognostic significance of VM in HCC. Kaplan–Meier analysis of overall survival based on VM in 201 patients. Kaplan–Meier survival curves showed that the presence of VM was associated with poor overall survival

We retrospectively evaluated the expression of HIF-1α and LOXL2 as well as the prevalence of VM among a cohort of 201 hepatocellular carcinoma specimens. The results showed that HIF-1α protein was highly expressed in 94 of 201 HCC sample tissues (46.76%; Fig. 1a and Table 1). LOXL2 protein was highly expressed in 102 of 201 HCC sample tissues (50.75%; Fig. 1a and Table 1). Elevated HIF-1α and LOXL2 expression was associated with an increase in tumor grade and VM (*p* < 0.05, Table 1). Finally, Kaplan–Meier survival analysis indicated that the HIF-1α and LOXL2 high expression groups have poor overall survival compared with the low expression groups (*p* < 0.05, Fig. 1b and c).

**Table 1:** Relationship between HIF-1α, LOXL2 and the cliniopathological features in hepatocellular carcinoma tissues

Variants	HIF-1α		*χ* ^2^	*P*-value	LOXL2		*χ* ^2^	*P*- value
	-	+			-	+		
Sex								
male	89	80	0.000	0.989	80	89	1.560	0.212
female	18	14			19	13		
Age (years)								
< 50	35	23	1.656	0.198	32	26	1.143	0.285
≥ 50	72	71			67	76		
Tumor size (cm)								
< 5	49	32	2.872	0.090	43	38	0.797	0.372
≥ 5	58	62			56	64		
Grade								
I + II	59	37	4.993	0.025*	55	41	4.750	0.029*
III + IV	48	57			44	61		
Metastasis								
NO	45	33	1.687	0.194	44	34	2.612	0.616
YES	62	61			55	68		
VM								
NO	89	64	6.270	0.012*	82	71	4.830	0.028*
YES	18	30			17	31		

*p <0.05

HIF-1α protein was highly expressed in 94 of 201 HCC sample tissues (46.76%). LOXL2 protein was highly expressed in 102 of 201 HCC sample tissues (50.75%). Elevated HIF-1α and LOXL2 expression was correlated with an increase in tumor grade and VM

Tube cavities lined with PAS-positive, CD31-negative tumor cells and red blood cells observed within the cavities were considered VM channels (red arrows, Fig. 1d). Channels positive for both PAS and CD31 were defined as EDVs (green arrows, Fig. 1d).

In 48 specimens (23.88%), VM was observed. Notably, our results demonstrated that the presence of VM correlates with the tumor grade, metastasis, and poor prognosis (*P* <0.05, Table 2 and Fig. 1e).

**Table 2:** The correlation of VM with the clinicopathological parameter of HCC

Variants	VM			
	negative	positive	*χ* ^2^	*P* -value
Sex				
Male	131	38	1.137	2.286
Female	22	10		
Age				
< 50	39	19	3.535	0.060
≥ 50	114	29		
Tumor size(cm)				
> 5	78	3	30.386	**
≤ 5	75	45		
Grade				
I + II	88	8	24.437	**
III + IV	65	40		
Metastasis				
NO	77	1	35.810	**
YES	76	47		

** *P* <0.01

In 48 specimens (23.88%), VM was observed. Notably, our results demonstrated that the presence of VM correlates with the tumor grade, metastasis, and poor prognosis

To understand the clinical relevance of HIF-1α and LOXL2, we also explored the relationship between HIF-1α and LOXL2 by conducting a correlation analysis of HIF-1α and LOXL2 in 201 HCC patients. The data showed that HIF-1α expression was positively correlated with LOXL2 in the HCC samples (Table 3).

**Table 3:** Relationship between HIF-1α and LOXL2 in hepatocellular carcinoma tissues

Group	HIF-1α	LOXL2
HIF-1α	__	*r* =0.1990
		*P* = 0.004**
LOXL2	__	__

**p <0.05

HIF-1α expression was positively correlated with LOXL2 in the HCC samples

### HIF-1α significantly regulates LOXL2 expression in hepatocellular cells

**Fig. 2:**
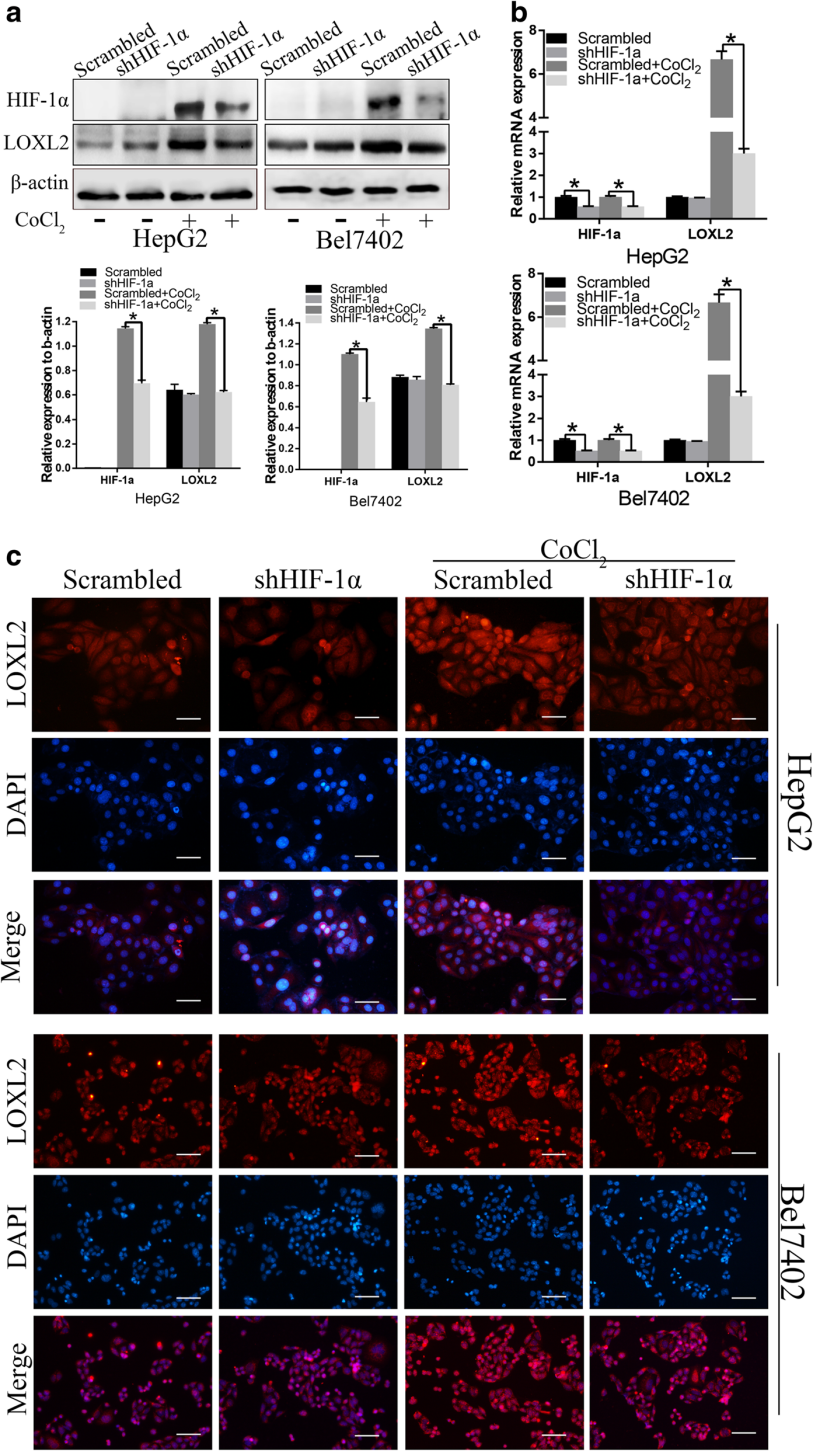
HIF-1α promotes LOXL2 expression was demonstrated by downregulation of HIF-1α with CoCl_2_ treatment in HepG2 and Bel7402 cells. **a** Western blot and **b** qRT-PCR results showed that knockdown of HIF-1α induced down-regulation of LOXL2 expression with CoCl_2_ treatment. **c** Immunofluorescence staining. Overexpression of HIF-1α increased the protein expression of LOXL2 and HIF-1α silencing expression inhibited LOXL2 expression (bar,50um). *P < 0.05

To confirm that HIF-1α induces LOXL2 expression in an HCC cell model, we treated HepG2 and Bel7402 HCC cells that stably expressed short hairpin RNA (shRNA) against HIF-1α with the hypoxia-mimetic agent CoCl_2_ (150 μM) for 48 h. The protein and mRNA expression levels of HIF-1α and LOXL2 of these two cell lines were assessed by Western blot and qRT-PCR, respectively, and the results showed that LOXL2 expression was up-regulated by HIF-1α, while HIF-1α knockdown ameliorates LOXL2 up-regulation in HepG2 and Bel7402 cells (Fig. 2a and b). Furthermore, immunofluorescence revealed that high LOXL2 expression occurs following HIF-1α overexpression. Conversely, cells decreased-expressing HIF-1α demonstrated loss of LOXL2 expression (Fig. 2c). The results demonstrated that LOXL2 expression was induced by HIF-1α.

### Changes in LOXL2 expression counteracted the aggressive phenotype induced by HIF-1α

**Fig. 3:**
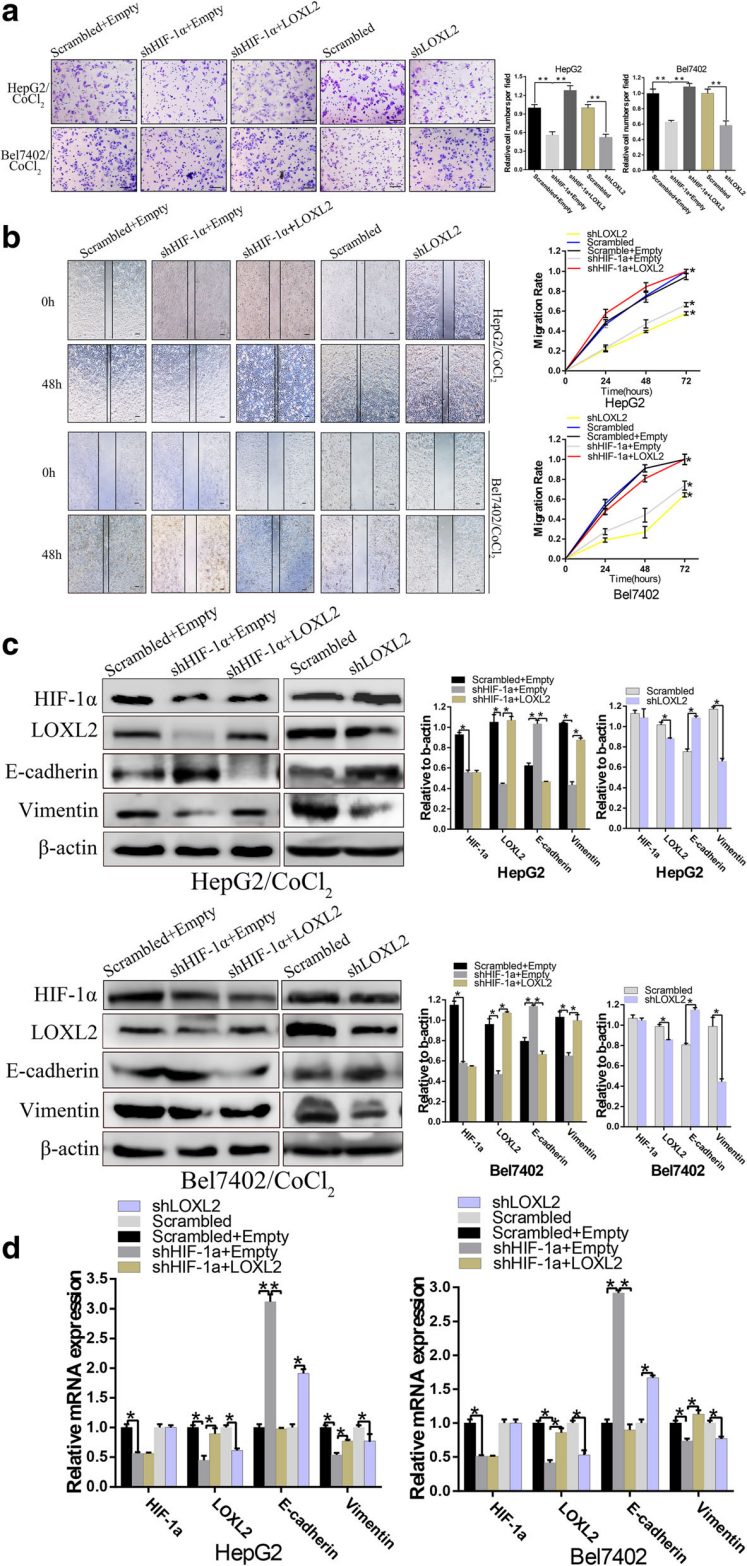
High HIF-1α expression promotes cell invasion, migration and EMT by regulating LOXL2 expression. **a** and **b**, The invasion and migration ability of HCC cells were decreased following HIF-1α knockdown and increased by LOXL2 overexpression; while shLOXL2 decreased invasion and migration ability, all groups treated with CoCl_2_ (a, ×100; bars, 100 μm) and (b,×40; bars, 100 μm). **c** and **d**, HepG2 and Bel7402 cells treated with CoCl_2_ were cotransfected with shHIF-1α and LOXL2 or the control vector and then western bolt and qRT-PCR assays were used to test the restoration of LOXL2 protein by LOXL2 plasmids in the presence of shHIF-1α. While HepG2 and Bel7402 cells were transfected with shLOXL2 plasmids or the control vector and then western bolt and qRT-PCR assays were used to test the restoration of LOXL2 protein by shLOXL2 in the presence of high HIF-1α expression. At the same time, expression of the epithelial protein E-cadherin and the mesenchymal protein vimentin in shHIF-1α transfected HepG2 and Bel7402 cells and LOXL2 stably transfected HepG2 and Bel7402 cells was detected by Western blot. qRT-PCR was used to detect mRNA expression. β-actin and GAPDH were used as loading controls. Error bars represent SD and *P < 0.05, **P < 0.01

Previous observations demonstrated that HIF-1α and LOXL2 exert similar effects on the aggressive phenotypes in HCC cells and that HIF-1α regulates LOXL2 expression. Based on these results, we performed a rescue assay to assess whether the effects of HIF-1α on HCC cells are mediated by LOXL2 expression. Cells were co-transfected with LOXL2 and the HIF-1α short hairpin plasmid, and overexpression of LOXL2 was confirmed to rescue the decrease in the protein and mRNA levels of LOXL2 caused by shHIF-1α expression and treatment with CoCl_2_ (Fig. 3c and d). As expected, restoring LOXL2 expression mostly blocked the inhibitory influence of HIF-1α knockdown on migration and invasion (Fig. 3a and b). Cells transduced with shLOXL2 plasmid were treated with CoCl_2_, and the low LOXL2 expression was confirmed to prevent the increase in the protein and mRNA levels of LOXL2 caused by HIF-1α (Fig. 3c and d). Ectopic LOXL2 expression counteracted the increased migration and invasion of HCC cells induced by HIF-1α (Fig. 3a and b).

EMT is an important cellular process during tumor invasion and migration, and the tumor cells that exhibit the ability to undergo EMT also have a greater ability to invade and metastasize; thus, EMT-related indexes were evaluated using Western blotting and quantitative RT-PCR. The results confirmed that elevated E-cadherin expression and down-regulation of vimentin, a mesenchymal marker, in HepG2 and Bel7402 cells treated with CoCl_2_ following HIF-1α knockdown. Conversely, cells overexpressing LOXL2 demonstrated a loss of E-cadherin and up-regulation of vimentin expression (Fig. 3c and d). At the same time, cells with LOXL2 knockdown that were treated with CoCl_2_ exhibited increased E-cadherin expression and down-regulation of vimentin. The gray analysis showed that these differences were statistically significant (Fig. 3c and d).

### HIF-1α promoted LOXL2-induced VM

**Fig. 4:**
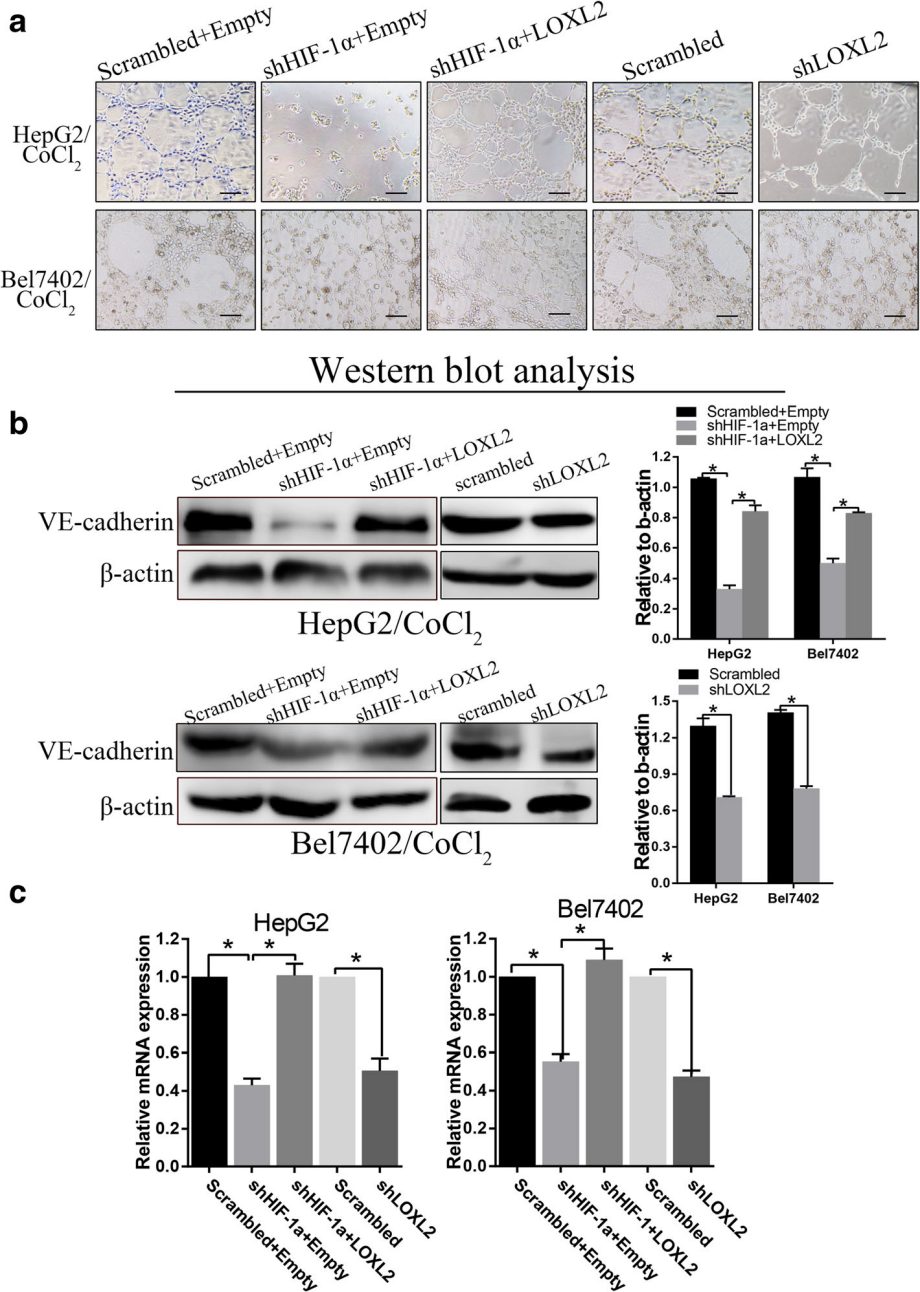
HIF-1α promoted VM formation by regulating LOXL2 expression. **a** Effects of HIF-1α and LOXL2 on the tube formation abilities of the HCC cell lines. **b** and **c** The protein and mRNA levels of VE-cadherin in HepG2 and Bel7402 cells, as analyzed by Western blotting and qRT-PCR. β-actin and GAPDH were used as loading controls. Original magnification: 100×, bar100μm. Error bars represent SD and *P < 0.05

To investigate the function of LOXL2 in HIF-1α-induced VM, we used a Matrigel-based tube formation assay. HIF-1α silencing in HCC cells inhibited VM in Matrigel, whereas LOXL2 overexpression facilitated the development of VM tubes. Treating cells with CoCl_2_ resulted in the formation of tube-like structures on the surface of the Matrigel, whereas loss of LOXL2 triggered the disappearance of these structures (Fig. 4a). The expression of vascular endothelial-cadherin (VE-cadherin), a marker of vascular mimicry (VM), was also explored by Western blot and qRT-PCR. The results showed that HIF-1α down-regulation can suppress VE-cadherin protein expression, while VE-cadherin protein expression was increased when LOXL2 was overexpressed. In addition, cells transfected with the LOXL2 short hairpin plasmid had lower VE-cadherin expression than control cells (Fig. 4b and c).

### The results of the genome-wide analysis of three groups of HCC cells

**Fig. 5:**
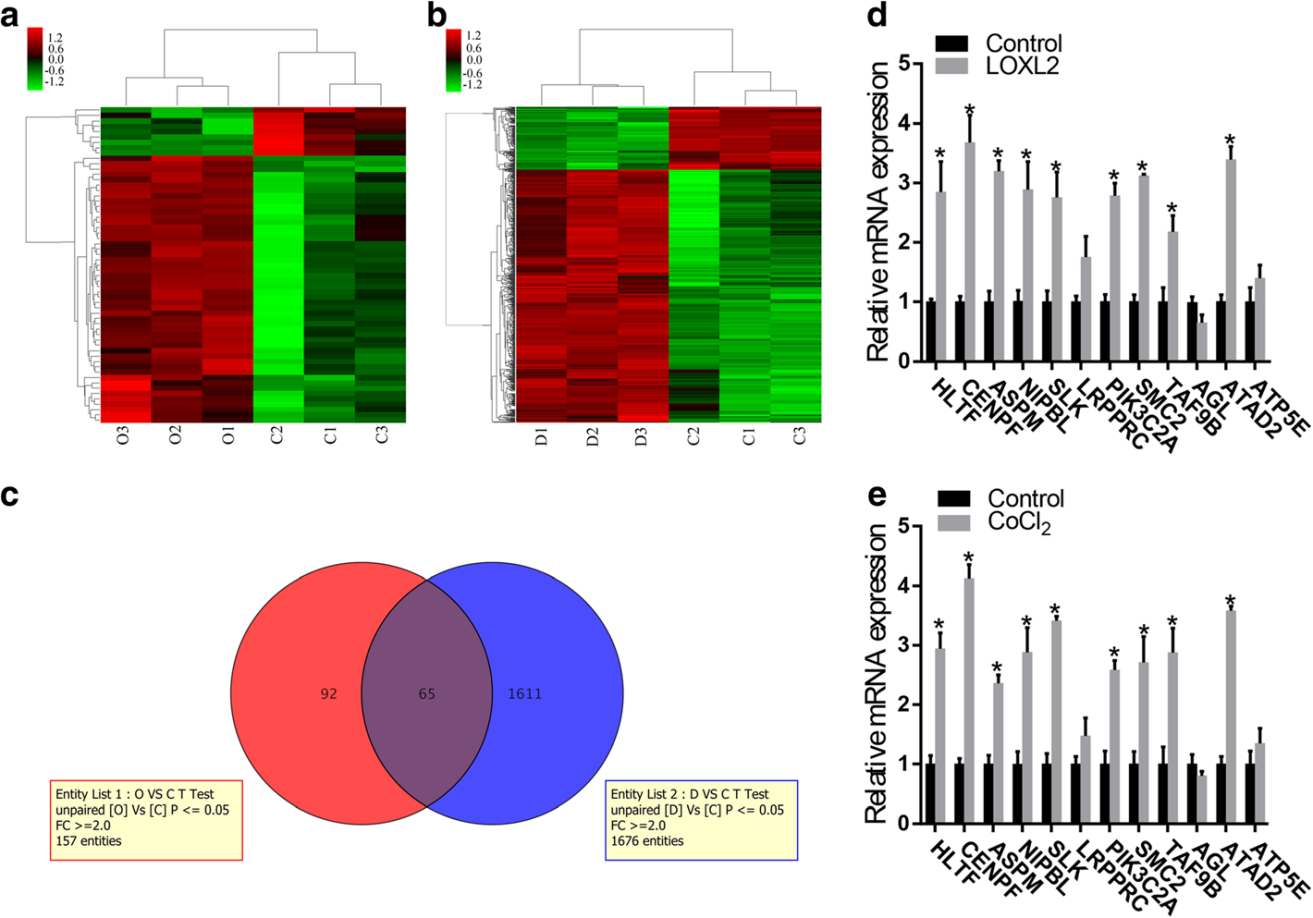
The results of the microarray among HepG2-LOXL2, HepG2-CoCl_2,_ and HepG2-Control cells. **a** A cluster analysis showed 70 differentially expressed genes between LOXL2 and Control group, including 48 upregulated genes and 22 downregulated genes(O represents LOXL2 group and C represents Control group). **b** The gene expression analysis showed 1059 differentially expressed genes between CoCl_2_ and Control group, including 771 upregulated genes and 288 downregulated genes(D represents CoCl_2_ group and C represents Control group). (genes with a fold change ≥2 and a P-value (*t*-test) <0.05 were collected). **c** The results of venn analysis between LOXL2 vs Control and CoCl2 vs Control. **d** qRT-PCR identified changes in expression levels between LOXL2 and Control group. **e** qRT-PCR was used to to validate the microarray results between CoCl_2_ and Control group.*P < 0.05

Next, we assessed the mRNA expression profiles in hepatocellular carcinoma HepG2 cells (named Control group), HepG2 cells transfected with plasmid LOXL2 to upregulate LOXL2 expression (named LOXL2 group), HepG2 cells treated with CoCl_2_ (named CoCl_2_ group). Comparison results showed that 70 genes were differently expressed between LOXL2 group and Control group, including 48 upregulated genes and 22 downregulated genes (Fig. 5a). 1059 genes showed differential expression between CoCl_2_ group and Control group, including 771 upregulated genes and 288 downregulated genes (Fig. 5b). Next, we analyzed these differently expressed genes whose fold change ≥ 2 in three groups. Moreover, we conducted venn analysis between LOXL2vsControl and CoCl_2_vsControl to make further study on LOXL2 induced by hypoxia. The results of venn analysis indicated 65differentially expressed genes (DEGs) that may associate with LOXL2 promoted by hypoxia (Fig. 5c). Among the 65 genes, 12 genes, including HLTF, CENPF, ASPM, NIPBL, SLK, LRPPRC, PIK3C2A, SMC2, TAF9B, AGL, ATAD2, ATP5E, have been considered as cancer-related genes by reviewing other researchers’ studies in PubMed. Then quantitative real-time PCR was carried out to validate the changes in expression levels of the 12 genes (Fig. 5d-e). Among the 12 genes, the expression of HLTF, CENPF, ASPM, NIPBL, SLK, PIK3C2A, SMC2, TAF9B and ATAD2 were consistent with the results of microarray analysis and all these genes have an upregulated tendency in HepG2 cells transfected with plasmid LOXL2 or treated with CoCl_2_. And the expression of AGL, LRPPRC and ATP5E have no change in LOXL2 group and CoCl_2_ group compared to Control group.

**Fig. 6:**
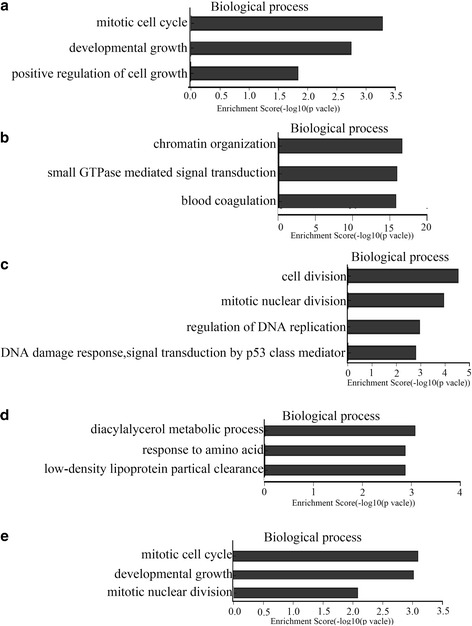
Gene Ontology (GO) analysis of biological process of genes which were upregulated and downregulated and their enrichment score. **a** GO analysis of biological process of genes, which were upregulated, associated with our study between LOXL2 and Control group. **b** GO analysis of biological process of genes which were downregulated between LOXL2 and Control group. **c** GO analysis of biological process of genes, which were upregulated, associated with our study between CoCl_2_ and Control group. **d** GO analysis of biological process of genes which were downregulated between CoCl_2_ and Control group. **e** GO terms of biological process of genes which were upregulated associated with our present study between LOXL2 vs Control and CoCl2 vs Control

In order to make functional interpretation for the gene expression changes, GO analysis was performed. Among the upregulated genes between LOXL2 group and Control group, we found several GO terms from the top 20 GO terms of biological process which may relate to our research, such as mitotic cell cycle, developmental growth and positive regulation of cell growth (Fig. 6a). Meantime, the biological process of GO analysis was applied for the downregulated DEGs in LOXL2 group, including chromatin organization, small GTPase mediated signal transduction and blood coagulation et al (Fig. 6b).

Following GO analysis for up-and downregulated DEGs in CoCl_2_ group, significant GO terms of biological process were collected. For upregulated DEGs, cell division, mitotic nuclear division, regulation of DNA replication and DNA damage response, signal transduction by p53 class mediator from the top 20 GO terms of biological process may relate to our research (Fig. 6c). For downregulated DEGs, GO analysis of biological process such as diacylglycerol metabolic process, response to amino acid and low-density lipoprotein particle clearance may not relate to cancer (Fig. 6d). In addition, the biological process of GO analysis of upregulated DEGs between LOXL2vsControl and CoCl2vsControl, such as mitotic cell cycle, developmental growth, stem cell maintenance and mitotic nuclear division may associate with our research (Fig. 6e and Additional file 1: Table S4). However, the GO analysis of the downregulated DEGs identified biological process which were not cancer related and might not play roles in our study.

## Discussion

Here, we demonstrated that there exists an important regulatory axis in tumor hypoxic microenvironment involving elevated levels of LOXL2 induced by HIF-1α; this increase results in suppression of E-cadherin suppression and activation of vimentin and the subsequent promotion of EMT and VM, both of which ultimately contribute to tumor progression in HCC.

Tumor hypoxia is a well-known phenomenon. As tumor cells grow, their microenvironment becomes increasingly hypoxic. Tumors as small as 1–2 mm in diameter may show signs of hypoxia and may depend on angiogenesis for additional growth [17]. Under hypoxic conditions, a signaling pathway involving the crucial oxygen response regulator hypoxia-inducible factor (HIF) is activated. The α subunit of HIF-1 (HIF-1a) is a well-established mediator in the cancer response to hypoxia [3]. The potential role of HIF-1a in tumor development was first identified from its observed overexpression in a broad range of tumor types and its involvement in key aspects of tumor development. Independent of any specific mechanism, HIF-1a overexpression has been associated with an unfavorable prognosis in most cancers because it activates genes that play a role in promoting cancer metabolism, angiogenesis, invasion, maintenance of stem cell pools, cellular differentiation, genetic instability, and metastasis [18–22]. In our present study, the results of the immunohistochemical staining demonstrated that HIF-1α is highly expressed in HCC specimens. Furthermore, HIF-1α accumulation has been associated with VM and poor patient survival.

Lysyl oxidase family members, in particular lysyl oxidase-like 2 (LOXL2), are known to regulate the process of EMT and thus promote tumor progression [10, 23–26]. Previous studies have indicated that LOXL2 is regulated by HIF1-α and is also a direct HIF1-α target gene [11, 27]. Our immunohistochemical staining data are consistent with these reports, as the pattern of HIF-1α expression mirrors the pattern of LOXL2 in HCC tissues. We used CoCl_2_ and HIF1-α knockdown to confirm that HIF1-α can induce LOXL2 expression in hepatocellular carcinoma cells in vitro.

Hypoxia can also induce the epithelial-mesenchymal transition (EMT), which is characterized by the loss of cell junctions and the acquisition of migratory behavior [28]. By promoting HIF1-α expression, a hypoxic tumor microenvironment can induce EMT, thus enhancing the tumor’s invasive and migratory abilities [29]. We proposed that HIF1-α may facilitate aggressive phenotypes by increasing LOXL2 expression. shHIF1-α and LOXL2 overexpression plasmids were co-transfected into HCC cells. We found that LOXL2 overexpression can rescue the inhibitory influence of shHIF1-α on migration and invasion. In addition, we transfected shLOXL2 plasmids into HCC cells and found that shLOXL2 can rescue the increased migratory and invasive influence caused by HIF1-α overexpression. These results prove that HIF1-α affects the migration and invasion of HCC cells partially by regulating LOXL2 expression.

EMT is closely related to an aggressive tumor phenotype in hepatocellular carcinoma [30]. In the present study, stable knockdown of HIF-1α in HepG2 and Bel7402 cells increased E-cadherin levels, while vimentin levels were decreased. Conversely, LOXL2 overexpression decreased E-cadherin levels and increased vimentin levels. In addition, transfection of short hairpin RNA specific to human LOXL2 blocked the activity of HIF-1α. The correlation between HIF1-α and EMT factors may explain the increased invasiveness and migration of HCC cells. In conclusion, these results demonstrate that HIF1-α affects migration and invasion by regulating LOXL2 expression.

Vasculogenic mimicry (VM) was first reported in highly aggressive uveal melanoma in 1999 [31]. Vasculogenic mimicry (VM) is an alternative method of supplying blood independent of endothelial vessels; this process refers to the formation of tumor cell-lined vessels and is associated with tumor invasion, metastasis and poor cancer patient prognosis [32]. VM has been found in HCC samples, and studies have reported that VM is associated with metastasis in HCC and can also result in a shorted overall survival [7]. The double-staining results demonstrated that the presence of VM correlates with the tumor grade, metastasis, and poor prognosis. Previous studies from our laboratory have demonstrated that hypoxia could promote VM. HIF-1α is a crucial factor in VM [7, 29]. VE-cadherin was one of the first molecules identified to promote VM in aggressive melanoma [31]. In addition, HIF1-α also modulates VM by stimulating VE-cadherin [33]. We found that HIF1-α could induce EMT, which can contribute to tumor cell plasticity. Our study indicated that increased VM was observed after inducing EMT in vitro. However, blocking LOXL2 could inhibit VM formation.

Furthermore, to understand the mechanism how cancer cell-derived LOXL2 can regulate HCC progression in hypoxic tumor microenvironment, we performed microarray analysis. Microarray analysis was conducted in HCC cells after upregulation of LOXL2. The results confirmed that cancer cell-derived LOXL2 affected genes expression in HCC cells. Abnormal proliferation and growth are characteristics of malignant tumors. It has been reported that downregulation of LOXL2 in HaCa4 and CarB cells can inhibit cells growth and proliferation [34]. The GO analysis of biological process about the upregulated DEGs indicted that LOXL2 may associate with mitotic cell cycle and cell growth in our study. Moreover, GO analysis of biological process between CoCl_2_ group and Control group showed that hypoxia can influence cell division and cell cycle. Therefore, our results demonstrated that LOXL2 overexpression or hypoxia-induced cell proliferation and division may involve in HCC aggressiveness.

Further GO analysis of biological process about the upregulated DEGs between LOXL2 vs Control and CoCl_2_ vs Control indicated that hypoxia may affect biological process of tumor, such as mitotic cell cycle, developmental growth, which were also impacted by upregulating LOXL2 expression. Therefore, these results suggested these biological function related to cancer aggressiveness might be affected via LOXL2 overexpression or the upregulation of LOXL2 induced by hypoxia.

Centromere protein F (CENPF) was identified as one significantly upregulated gene in our microarray analysis and also belonged to the 65 DEGs identified by the venn analysis. And the results of qRT-PCR indicated that the expression of CENPF was the highest in LOXL2 group and CoCl_2_ group compared to Control group. This gene encodes a protein that associates with the centromere-kinetochore complex and chromosomal segregation during mitosis. Previous studies have demonstrated that the upregulation of CENPF may play a role in the regulation of cell division and may be used as proliferation marker of malignant cell growth in clinical practice due its localizations in the cell cycle [35–37]. It has been reported that the overexpression of CENPF associated with poor prognosis in hepatocellular carcinoma, breast cancer, colorectal gastrointestinal stromal tumors, esophageal squamous cell carcinoma and prostate cancer [38–42]. Silencing CENPF can decrease the ability of HCC cells to proliferate, form colonies and induce tumor formation in nude mice [38]. Our data suggests that the upregulation of CENPF following LOXL2 overexpression or hypoxia may play a critical role in driving HCC development.

ATPase family, AAA domain containing 2 (ATAD2), another upregulation DEGs, is a member of the AAA + ATPase family. The qRT-PCR displayed that the expression of ATAD2 was the second-highest in LOXL2 group and CoCl_2_ group compared to Control group. ATAD2, the predicted protein product which contains both a bromodomain and an ATPase domain, maps to chromosome 8q24 in a region that is frequently found amplified in cancer [43]. The overexpression of ATAD2 has been reported in multiple solid tumors in humans, such as breast cancer, cervical cancer, glioma, hepatocellular carcinoma, ovarian carcinomas and gastric cancer [44–49]. ATAD2 has been suggested to play important role in tumorigenesis through regulating cell differentiation, proliferation, metastasis, apoptosis and cell cycle [46, 48, 50]. The suppression of ATAD2 expression elicited anti-tumor functions, including inhibition of HCC cell proliferation, migration, invasion and ATAD2 suppression in subcutaneous HCC xenografts delayed tumor cell growth, accompanied by apoptosis induction [51]. Taken together, our data suggests that LOXL2 overexpression or hypoxia may affect HCC progression by promoting ATAD2 expression.

## Conclusion

In summary, this study demonstrated that HIF1-α promotes LOXL2 expression and subsequently induces EMT and VM in HCC cells, thus promoting HCC progression. This study highlights the potential therapeutic value of targeting LOXL2 for suppression of HCC metastasis and progression. And how cancer cell-derived LOXL2 can regulate HCC progression in hypoxic tumor microenvironment gained better understand.

## Additional file

Additional file 1: Table S4. The GO analysis of biological process of genes which were upregulated between LOXL2 vs Control and CoCl_2_ vs Control. Legend: the GO terms of biological process of genes which were upregulated between LOXL2 vs Control and CoCl_2_ vs Control. (CSV 30 kb)

## Abbreviations

ECM: Extracellular matrix
EDVs: Endothelial depended vessels
EMT: Epithelial-mesenchymal transition
HCC: Hepatocellular carcinoma
HIF-1α: Hypoxia-inducible factor-1α
LOXL2: Lysyl oxidases like 2
VM: Vasculogenic mimicry
